# Ibuprofen and sila-ibuprofen: polarization effects in the crystal and enzyme environments

**DOI:** 10.1107/S2052520621009379

**Published:** 2021-11-12

**Authors:** Florian Kleemiss, Pim Puylaert, Daniel Duvinage, Malte Fugel, Kunihisa Sugimoto, Jens Beckmann, Simon Grabowsky

**Affiliations:** a Universität Bern, Departement für Chemie, Biochemie und Pharmazie, Freiestrasse 3, 3012 Bern, Switzerland; b Universität Regensburg, Fakultät für Chemie und Pharmazie, Universitätsstr. 31, 93053 Regensburg, Germany; c Universität Bremen, Fachbereich 2 - Biologie/Chemie, Institut für Anorganische Chemie und Kristallographie, Leobener Str. 7, 28359 Bremen, Germany; d Japan Synchrotron Radiation Research Institute/Diffraction & Scattering Division, 1-1-1 Kouto, Sayo-cho, Sayo-gun, Hyogo 679-5198, Japan; e Institute for Integrated Cell-Material Sciences (iCeMS), Kyoto University, Yoshida-Ushinomiya-cho, Sakyo-ku, Kyoto 606-8501, Japan

**Keywords:** quantum crystallography, ibuprofen, cyclooxygenase, interaction density, electrostatic potential, sila-ibuprofen, polarization

## Abstract

Sila-ibuprofen is a new potential nonsteroidal anti-inflammatory drug which deviates from its parent ibuprofen in terms of the electrostatic potential around the carbon/silicon-switched C/Si—H group. Therefore, sila-ibuprofen is more water soluble and has a lower melting enthalpy. However, its binding and inhibition properties of cyclooxygenases appear to be very similar to regular ibuprofen. Therefore, in this study, intermolecular interactions and interaction densities of both ibuprofen and sila-ibuprofen in their biologically active forms, *i.e.* deprotonated and as the pure *S*-enantiomers are investigated. Quantum-crystallographically refined salts with argininium and 1-phenyl­ethan-1-amoninium (PEA) counter-cations as crystalline models of the interactions with the guanidine functional group of arginine inside cyclooxygenases are presented. The similarities and differences between the polarization of ibuprofen and sila-ibuprofen in the crystal, enzyme, solvent and isolated environments are discussed based on quantum-chemical calculations. For the explicit crystal and enzyme environments, specifically, molecular dynamics simulations starting from the crystal models were combined with QM/MM calculations.

## Abstract 2

Intermolecular interactions of the new drug candidate sila-ibuprofen are investigated in the crystal and in the enzyme cyclooxygenase-II.

## 1. Introduction

Ibuprofen is one of the most important and widely used drugs of humankind. It is a nonsteroidal anti-inflammatory drug (NSAID) used for pain relief, which was first synthesized and patented in 1961 (Halford *et al.*, 2012 ▸). It inhibits cyclo­oxygenase-II (COX-II), an enzyme that is produced by the body when tissue damage or inflammation occurs, and blocks the synthesis of prostaglandins from arachidonic acid (Prusakiewicz *et al.*, 2009 ▸). For pain relief, only the *S*-enantiomer of ibuprofen is active. The *R*-enantiomer can be converted into the *S*-enantiomer in the body by a racemase, so that the drug can be administered as a racemic mixture (Rainsford, 2015 ▸). The form of ibuprofen bonded to COX-II is the deprotonated anionic form.

Numerous crystal structures of ibuprofen and ibuprofenate have been published. A Cambridge Structural Database (CSD; Groom *et al.*, 2016 ▸) search (made in April 2021) returned 48 different crystal structures of neutral ibuprofen and 22 of deprotonated ibuprofenate. Some seminal and interesting crystallographic studies of the racemic version com­prise Connell (1974 ▸), Dudognon *et al.* (2008 ▸) and Walsh *et al.* (2003 ▸), and also neutron diffraction work in Shankland *et al.* (1997 ▸), experimental electron-density work in Bouhmaida *et al.* (2002 ▸) and high-pressure work in Ostrowska *et al.* (2015 ▸). The crystal structure of the enantiomerically pure *S*-enantiomer is also known (Freer *et al.*, 1993 ▸; Hansen *et al.*, 2003 ▸), but not that of the *R* form. Additionally, the *S*-enantiomer of ibuprofen can either crystallize in its neutral form with different molecules as a cocrystal [protonated, for example, with nicotinamide (Berry *et al.*, 2008 ▸) or pyridine (Chen *et al.*, 2010 ▸)] or in its deprotonated *S*-ibuprofenate form as a salt with counter-cations. As outlined in Springuel *et al.* (2014 ▸), it makes a difference to the properties of the crystal whether *S*-ibuprofen is cocrystallized or forms a salt. Since ibuprofen is deprotonated inside the enzyme, ibuprofenate salt crystal structures are most relevant for this study, in which we aim for a com­parison of the enzyme and crystal environments. Most examples of ibuprofenate salts com­prise ammonium counter-cations where the related amine is basic enough to depro­ton­ate the carboxylic acid group of ibuprofen (Kumar *et al.*, 2017 ▸; Lemmerer *et al.*, 2010 ▸; Rehman *et al.*, 2018 ▸; Ma *et al.*, 2019 ▸). In this study, we will use *R*- or *S*-1-phenylethan-1-amine (PEA) to produce salts of ibuprofen and sila-ibuprofen (Lemmerer *et al.*, 2010 ▸; Molnár *et al.*, 2009 ▸), and also report on the new crystal structure of argininium ibuprofenate. The resulting ammonium (sila-)ibuprofenate interactions in the crystals serve as models of the interactions with the guanidine functional group of arginine inside cyclooxygenase-II.

**Figure 1:**
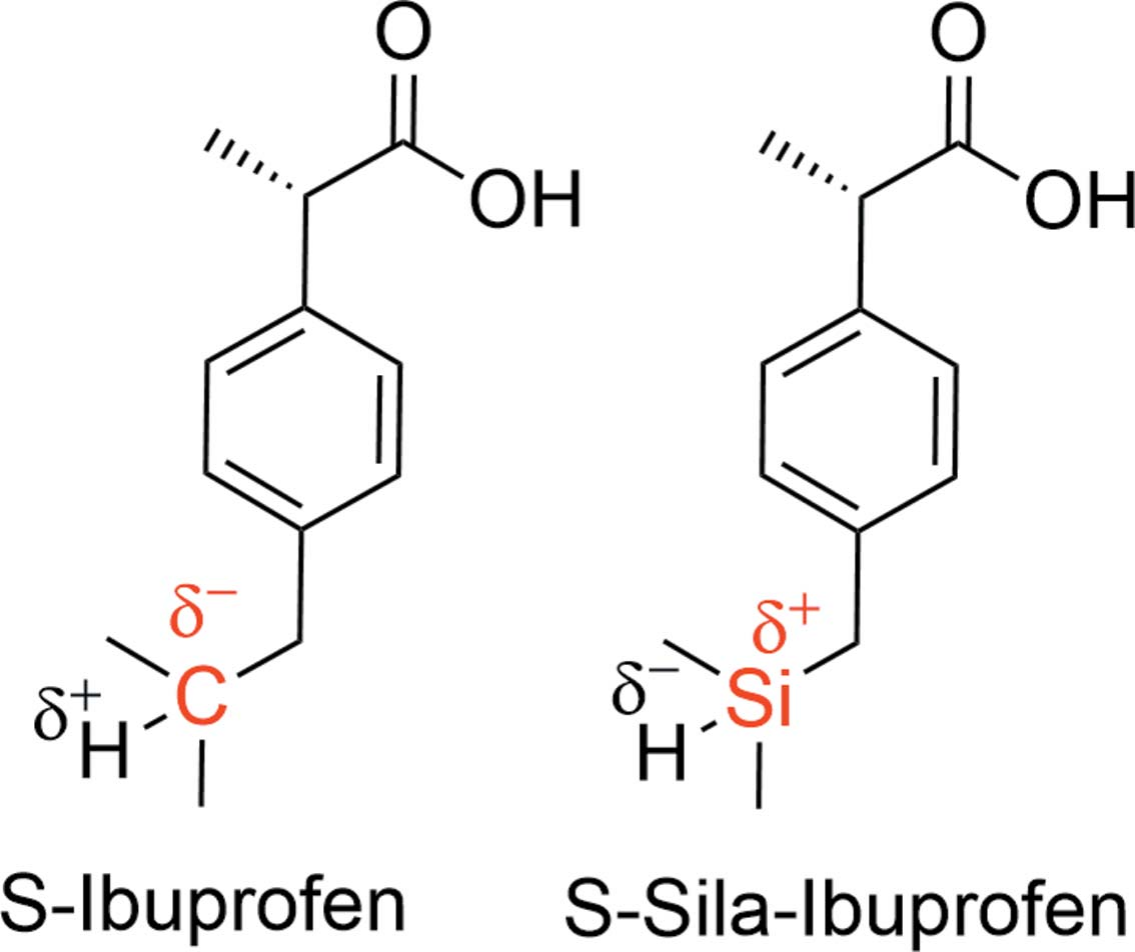
Ibuprofen and sila-ibuprofen as their biologically active *S*-enantiomers.

Recently, we presented a new derivative of ibuprofen, in which the tertiary C atom is replaced by an Si atom to yield sila-ibuprofen (see Fig. 1 ▸) (Kleemiss *et al.*, 2020 ▸). Sila-ibuprofen was found to be a bioisoster of ibuprofen, but with different physical properties, such as a lower melting enthalpy and better solubility in aqueous media. The differences in these properties can be understood because carbon/silicon exchange leads to the umpolung of the related C/Si—H bond (Fig. 1 ▸), which in turn results in a different electrostatic potential and molecular dipole moment (Kleemiss *et al.*, 2020 ▸). However, since a different electrostatic potential should also influence the biological recognition process and the polarization of the molecule in a biological environment, we will study the polarization of both ibuprofen and sila-ibuprofen in different environments in more detail here.

The crystalline environment has been used for a long time to simulate and mimic both the conformation and the polarization of small pharmaceutically active ingredients in biological environments (Klebe, 1994*a* ▸,*b* ▸; Pascard, 1995 ▸; Luger, 2007 ▸). However, the extent to which this assumption is true has only rarely been investigated (Mladenovic *et al.*, 2009 ▸; Grabowsky *et al.*, 2013 ▸). Only very recently, we have used the concept of interaction density defined in crystallography (Krijn *et al.*, 1988 ▸; Spackman *et al.*, 1999 ▸; De Vries *et al.*, 2000 ▸; Dittrich & Spackman, 2007 ▸) to evaluate this question in depth for a model com­pound of the cystein protease inhibitor E64c (loxistatin acid) (Kleemiss *et al.*, 2021*b* ▸). Here we combine the techniques of quantum crystallography (Grabowsky *et al.*, 2017 ▸; Genoni *et al.*, 2018 ▸; Genoni & Macchi, 2020 ▸), molecular dynamics and QM/MM to investigate the question with respect to the ibuprofen/sila-ibuprofen pair.

## 2. Experimental details

### 2.1. Crystallization and structure determination

Ibuprofen was obtained commercially and sila-ibuprofen was prepared according to our recently reported procedure (Kleemiss *et al.*, 2020 ▸). The enantiomeric separation of both ibuprofen and sila-ibuprofen was attempted using a procedure reported in the literature, where either *R*- or *S*-1-phenylethan-1-amine (PEA) is added to a solution of racemic ibuprofen to form only salts of one of the enantiomers (McCullagh, 2008 ▸). The crystal structures of PEA salts with both *S*- and *R*-ibu­profen are reported in the literature (Lemmerer *et al.*, 2010 ▸; Molnár *et al.*, 2009 ▸). In this study, we added both *R*- and *S*-PEA to racemic solutions of ibuprofen and sila-ibuprofen in separate experiments and attempted to grow crystals from each of the four experimental setups. We could only obtain two different types of crystals. The crystallographic analysis showed (see below) that these two types belong to a mixed crystal of *ca* 20/80% *S*-/*R*-ibuprofen with *S*-PEA and to an *S*-sila-ibuprofen *R*-PEA salt crystal.

For the PEA sila-ibuprofenate salt, only crystals of very low quality could be obtained and these scattered very weakly. Therefore, both crystals were measured at SPring-8, beamline BL02-B1, at 25 K using a large curved image-plate detector and a wavelength of 0.3567 Å. More crystallographic and measurement details are given in Table 1 ▸.

**Figure 2:**
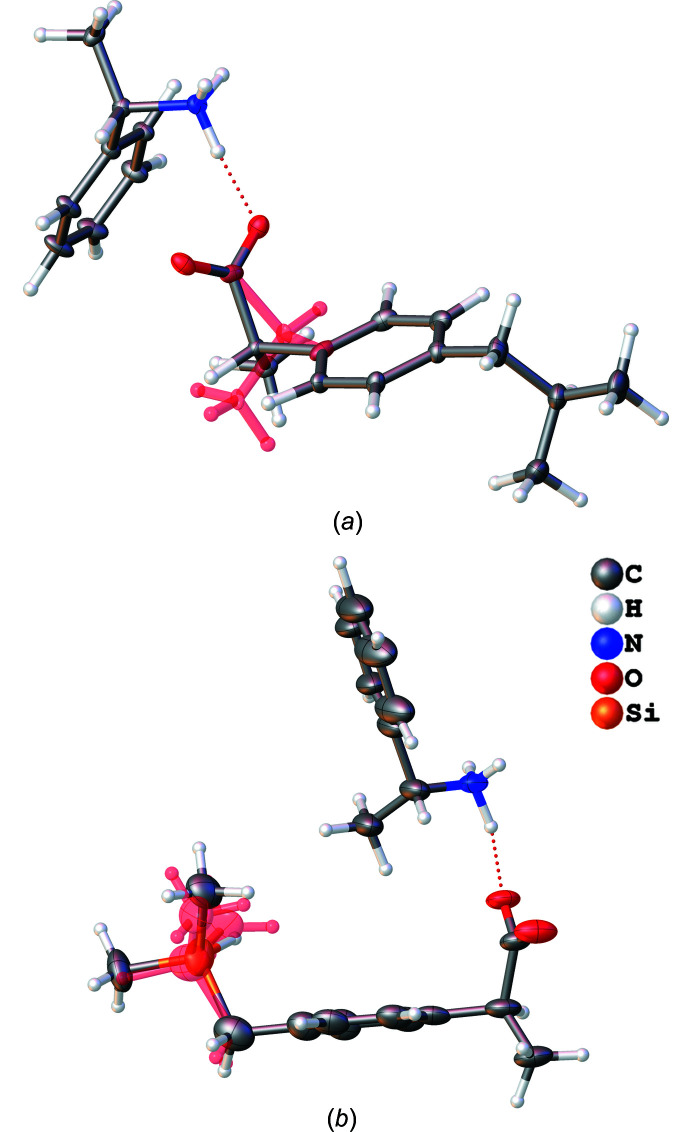
Visualization of the HAR structures of the ibuprofen (*a*) and sila-ibuprofen (*b*) salts formed with PEA. Atomic displacement parameters are drawn at the 50% probability level. Both disorder parts are shown for both structures. In the ibuprofen structure, the *R*-enantiomer has 81.2 (7)% occupancy. In sila-ibuprofen, only the *S*-enantiomer is found and the disorder of the methyl groups is about 52/48%. The salt of ibuprofen was formed with *S*-PEA, while the sila-ibuprofen salt was formed with *R*-PEA.

The structures were solved with *SHELXT* (Sheldrick, 2015 ▸) and refined using Hirshfeld Atom Refinement (HAR) (Capelli *et al.*, 2014 ▸; Jayatilaka & Dittrich, 2008 ▸; Woińska *et al.*, 2016 ▸) in its *NoSpherA2* implementation (Kleemiss *et al.*, 2021*a* ▸). A level of theory of PBE/def2-TZVPP was used during the wavefunction calculation in *ORCA* (Neese, 2012 ▸, 2018 ▸) as part of the HAR procedure. Anomalous dispersion values for the employed wavelength were used from the Sasaki tables (Sasaki, 1989 ▸). The final geometries are visualized in Fig. 2 ▸ and the refinement statistics are given in Table 1 ▸.

It was found that the enantiomeric separation was not com­plete for the ibuprofenate–PEA salt. A small amount of the other enantiomer was identified in the crystal structure, which could be modelled by a disorder refinement. Neighbouring ordered atoms were split but treated using constraints for identical positions and atomic displacement parameters, so that two types of scattering factors could be calculated with *NoSpherA2*, accounting for the different bonding situations adopted by these atoms. In the ibuprofenate–PEA salt, the occupancy of the *S*-enantiomer was refined to be 0.188 (7), which means there is 81.2 (7)% of the *R*-enantiomer in the crystal structure. Since enantiomerically pure PEA was used, the assignment of the absolute configuration to the disorder parts is chemically unambiguous. For the sila-ibuprofenate–PEA salt, only the *S*-enantiomer was found. However, there is a different disorder around the dimethylsilane functional group, which was treated by creating two parts that were rotated to match the residual density peaks.

Since both the quality and the resolution of the data set of the salt of sila-ibuprofen and PEA are low, not all H-atom positions could be refined freely. The H atoms of the CH_2_ group of sila-ibuprofen were given identical *U*_iso_ values in both parts and the methyl groups attached to the Si atom were also refined using a free variable to define their *U*_iso_ values. All C—H distances were fixed using the corresponding geometric constraints commands based on reported averaged distances from neutron diffraction experiments (Allen & Bruno, 2010 ▸). The N—H distance was refined freely and the Si—H distance of the silane functional group was fixed to the distance obtained from the crystal structure of racemic sila-ibuprofen according to Kleemiss *et al.* (2020 ▸). Crystallographic information files (CIFs) for both crystal structures can be obtained from the Cambridge Structural Database (CSD) under deposition numbers CCDC-2034508/9, and as supporting information with this article.

We note that a HAR of these data sets was only possible since the refinement of disorder and use of restraints, riding models and adjustable (*e.g.* *SHELX*-type) weighting schemes was enabled as part of recent *NoSpherA2* developments (Kleemiss *et al.*, 2021*a* ▸). Moreover, the nonspherical refinement model used in HAR sharpened the residual density features, so that the assignment of disorder com­ponents was facilitated. The occupation parameters became more precise, and the weighting scheme coefficients, as well as the *R* statistics, were lowered com­pared to the initial independent atom model refinement.

The PEA–ibuprofen and PEA–sila-ibuprofen structures could be understood as a model of the interaction of the small drug molecules with an amine function, similar to the binding with the guanidine functional group of arginine inside COX-II. To produce a more direct model of the interaction with COX-II, cocrystallization with arginine was carried out. Attempts at crystallization using sila-ibuprofen were unsuccessful and only yielded oils or crystals of the reactants, but, to the best of our knowledge, the argininium–ibuprofenate struc­ture we obtained is not yet known either. Further analysis of this crystal structure was not pursued, but a discussion and visualization is given in the supporting information.

### 2.2. Simulations and com­putations

The following environments around (sila-)ibuprofen were studied: isolated molecule/gas (**G**), solvation (**S**), crystal (**C**) and protein (**P**) models. Molecular dynamics simulations (MD) and QM/MM calculations were performed with *NAMD2* (Kalé *et al.*, 1999 ▸; Phillips *et al.*, 2005 ▸) and *ORCA* (Neese, 2012 ▸, 2018 ▸), which are directly interfaced to each other in the latest software versions. The protein reference environments (model **P**), including the MD and QM/MM calculations used in this study, are the COX-II com­plexes of both molecules as produced and analyzed in Kleemiss *et al.* (2020 ▸), starting from the ibuprofen–COX-II com­plex crystal structure reported in Orlando *et al.* (2015 ▸).

An MD simulation setup for model **C** of 11 × 11 × 11 unit cells, which corresponds to a cell size of *ca* 65 × 168 × 245 Å for PEA–ibuprofen and 75 × 140 × 260 Å for PEA–sila-ibuprofen, was constructed based on the symmetry of the experimental crystal structures. In both cases, only the structure (disorder com­ponent) with an *S*-configuration of the (sila-)ibuprofen unit was considered, as this is the biologically active one. A visualization of the simulated crystal fragment for PEA–ibuprofen is shown in Fig. S1 in the supporting information. The same force field parameters were used for ibuprofen and sila-ibuprofen as developed in Kleemiss *et al.* (2020 ▸). The PEA molecule was modelled here using a combination of parameters present in the CHARMM-type force field for methylammonium and phenylalanine, which were combined, and charges were adapted to result in an integer single positively charged entity while applying the fewest possible changes with regard to the original charges in CHARMM. The resulting parameters are given in Tables S1 and S2 in the supporting information. After initial minimization of the input structures, annealing starting at 60 K, was performed in 1 K steps of 1200 fs each until the production temperature of 300 K was reached. An additional 500 000 steps of equilibration were carried out prior to production runs, employing the following production settings: a Langevin-thermostat targeted at 300 K, an isotropic Langevin–Piston-barostat working at 1.01325 bar (1 bar = 10^5^ Pa) and a time step for integration of the equations of motion of 2 fs. A cutoff of 12 Å was used for Lennard–Jones interactions, switching at 10 Å to a smoothing function. The geometries of the pro­duction runs were saved every ps.

The MD was performed to obtain a measure of thermal fluctuation in the crystal to com­pare with the protein environment. This was visualized by means of the averaged noncovalent interaction index (aNCI) (Wu *et al.*, 2013 ▸). A molecule in the middle of the cluster was chosen and the aNCI was calculated on a second production run, fixing the position of two C atoms of the arene ring of the selected molecule after the equilibration run. In this way, the molecule was given the highest degree of flexibility while prohibiting translations which would com­promise the calculation of the aNCI. The so far unpublished software *cuQCT*, written by F. Kleemiss (Kleemiss, 2020 ▸), was used to calculate the aNCI in an accelerated way on graphics cards.

QM/MM minimizations starting from the geometries of the MDs were performed to obtain wavefunctions for the calculation of interaction densities. For model **C**, all the QM/MM minimizations yielded the same potential-energy minimum. For model **P**, several slightly differing QM/MM minima were obtained because the thermal fluctuations of the MDs were higher. Therefore, five geometry snapshots were taken during the last quarter of the MD production runs and five different QM/MM minimizations were performed. The resulting QM/MM geometries and electron densities in grid files were averaged. Additionally, geometry optimizations of the ibu­pro­fenate and sila-ibuprofenate anions were performed in a vacuum (model **G**) and using an implicit solvation model of water in *ORCA*, corresponding to model **S**. For the solvation model, the Conductor-Like Polarizable Continuum Model (CPCM) (Tomasi *et al.*, 2005 ▸) of water was used as implemented in *ORCA*. The level of theory for the calculations used was B3LYP/def2-TZVP within *ORCA*.

The first step towards the calculation of the interaction density was a single-point wavefunction calculation on a structure that was obtained by optimization in a given environment and subsequent com­putation of its electron density on a grid. This grid was chosen in an identical manner for both wavefunctions, once in the environment and once without the influence of the environment. Finally, the calculation of the difference between the grids yields the interaction density. These com­putations of grids were performed using *cuQCT*. By definition, the grid file without the effect, that is the one calculated from the molecule in the vacuum at the same geometry as in the respective environment, was subtracted from that with the effect, which is the one in models **P**, **C** and **S**. For more details on this procedure, see Kleemiss *et al.* (2021*b* ▸).

As similarity measures between two different electron-density distributions, we used the real-space *R* value (*R*_RS_) (Jones *et al.*, 1991 ▸) and the integrated number of electrons in the interaction density grid file (*N*_e_). 

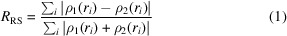




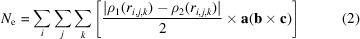

where **a**, **b** and **c** are the vectors defining the grid, and *i*, *j* and *k* are the indices used for each grid point. The difference or interaction density of a single molecule must sum to a value of 0 when the com­plete space is considered. Therefore, the number of electrons shifted refers to the absolute integrated values of differences divided by 2 for *N*_e_. This leads to valid ranges of [0,1] for *R*_RS_ and of [0,*N*] for *N*_e_, where *N* is the number of electrons in the molecule.

## 3. Results and discussion

### 3.1. Noncovalent interactions

**Figure 3:**
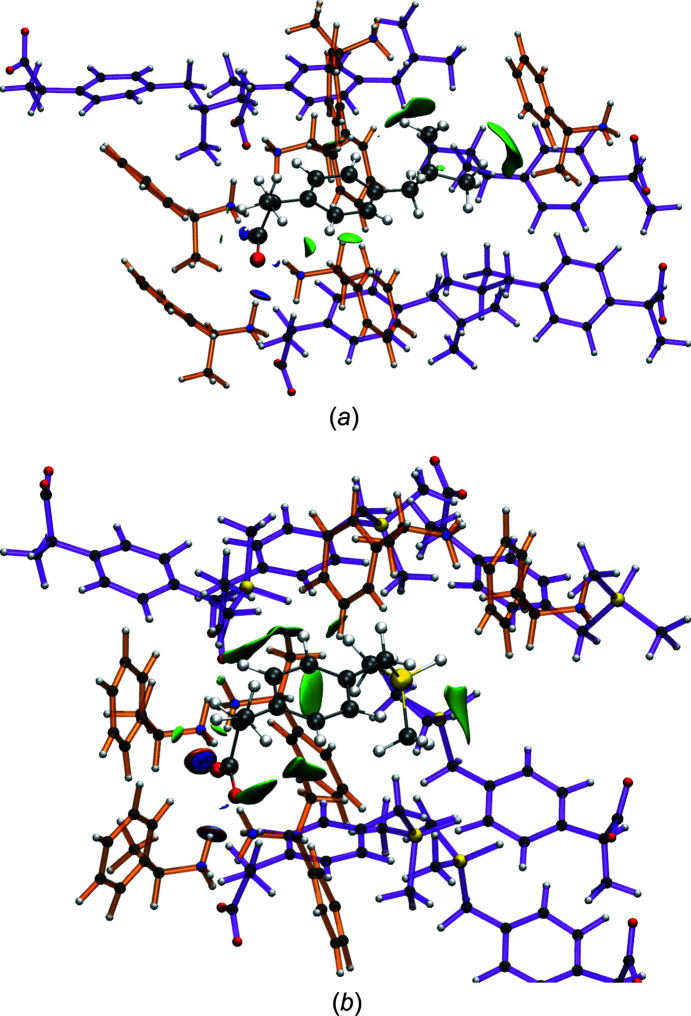
Plot of isosurfaces of the aNCI between (*a*) ibuprofen and (*b*) sila-ibuprofen, and the neighbouring molecules in the crystal. Blue-coloured isosurfaces refer to attractive (electrostatic) interactions, whereas green refer to weaker (dispersion) interactions. Neighbouring molecules are colour coded also, *i.e.* PEA has orange bonds and ibuprofen/sila-ibuprofen have purple bonds. Atoms were given the same colour code as the main molecule. The visualization was made using *VMD* (Humphrey *et al.*, 1996 ▸). Corresponding representations of the NCI in the static crystal structures of ibuprofen–PEA and sila-ibuprofen–PEA are given in the supporting information.

**Figure 4:**
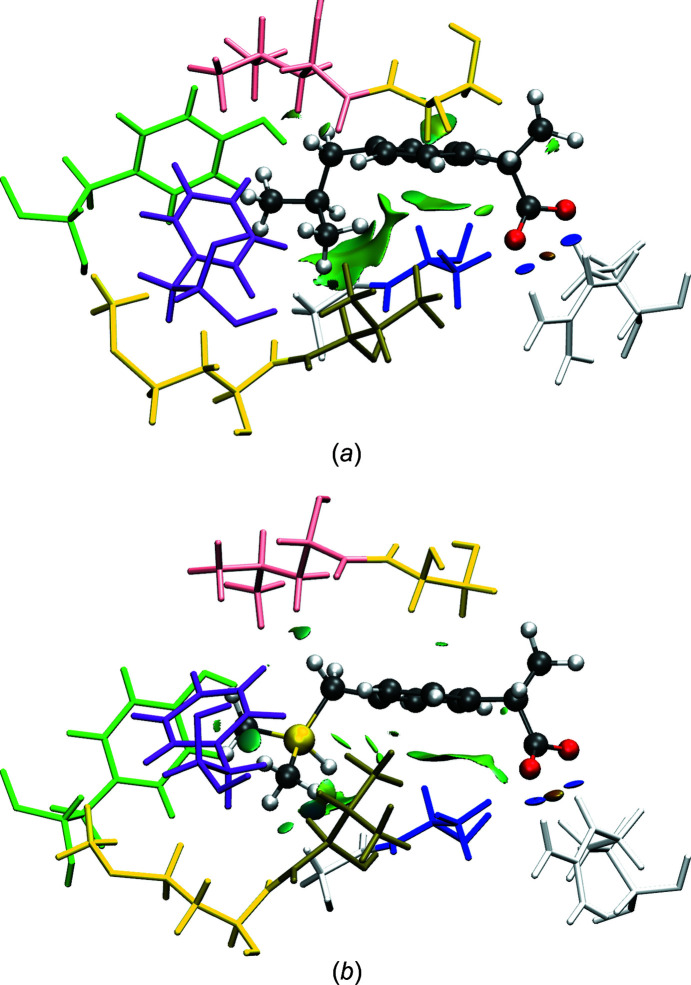
Plot of isosurfaces of the aNCI between (*a*) ibuprofen and (*b*) sila-ibuprofen, and the neighbouring molecules inside COX-II. For a better view of the isosurfaces, the orientation is rotated by about 180° com­pared to Fig. 3 ▸. Blue-coloured isosurfaces refer to attractive (electrostatic) interactions, whereas green refer to weaker (dispersion) interactions, while orange refers to repulsion. Neighbouring stick-style molecules are colour coded as follows: grey = Arg, blue–grey = GlyAla, yellow–brown = MetVal, purple = Phe, yellow–pink = SerLeu and green = Tyr. The visualization was made using *VMD* (Humphrey *et al.*, 1996 ▸).

A visualization of the aNCI of ibuprofen and sila-ibuprofen inside the PEA salt crystals is given in Fig. 3 ▸ and inside the enzyme COX-II in Fig. 4 ▸. When com­paring the aNCI of ibuprofen and sila-ibuprofen in the PEA salt crystals, similar patterns of interactions are observed. Hydrogen bonds with neighbouring amino residues around the carboxylate group and dispersion interactions below the arene ring and at the isobutyl group are the dominating features. The amino–carboxylate N—H⋯O hydrogen bond is similar to that of the guanidino–carboxylate interaction found in the active site of COX enzymes. The major difference is that in the PEA salt crystals the two O atoms interact with two different molecules, giving rise to three short N—H⋯O contacts (see left-hand side of Figs. 3 ▸(*a*) and 3 ▸(*b*) ▸, while the twofold hydrogen bond in the active site of COX links the ibuprofen molecules to one residue only *via* two short N—H⋯O contacts (see right-hand side of Figs. 4 ▸(*a*) and 4 ▸(*b*). These three contacts in the sila-ibuprofen–PEA structure have O⋯N distances of 2.65 (8), 2.63 (7) and 2.65 (8) Å averaged over the whole simulation time. In the case of the ibuprofen–PEA structure, the distances are 2.62 (7), 2.66 (9) and 2.60 (7) Å, respectively. The averaged values of the O⋯N distances for the protein environment, where the guanidino function is the interaction partner, are 2.66 (9) and 2.8 (2) Å in the case of ibuprofen inside COX-II. For sila-ibuprofen, the distances are 2.66 (9) and 2.66 (9) Å. Although some distances in the enzyme are larger and have higher standard deviations, the lengths and strengths of the hydrogen bonds are com­parable, as reflected in the plots of the aNCI.

Further remarkable resemblances between the aNCI plots of the PEA salts and in the active site of COX-II are found (com­paring Figs. 3 ▸ and 4 ▸): while the aNCI plot around the C—H/Si—H function is not dominating the picture, the interactions between the methyl groups attached to the carbon/silicon-switched position show big areas of dispersion interactions with neighbouring molecules. The hydrogen bonds in the vicinity of the carboxylate groups are the strongest interactions, as shown by their blue isosurfaces, coinciding with the observation of similar hydrogen-bond lengths discussed in the previous paragraph. In the case of ibuprofen–PEA, the interaction due to dispersion around the arene ring is lower in com­parison to sila-ibuprofen; this interaction is not very pronounced in the COX-II environment either. Since similar areas show interactions with the neighbourhood in both systems, it can be expected that the polarization of the electron density might also be similar. Therefore, an investigation of the interaction density is performed in the following section.

### 3.2. Interaction density

**Figure 5:**
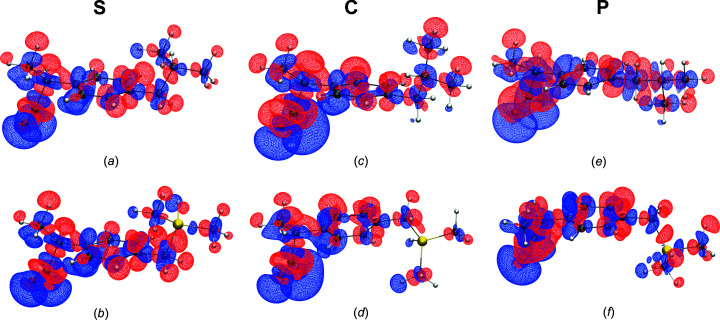
Plots of the interaction density isosurfaces at isovalue 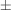 0.001 a.u. (blue = positive and red = negative) for ibuprofenate [parts (*a*), (*c*) and (*e*)] and sila-ibuprofenate [parts (*b*), (*d*) and (*f*)] in different environments: (*a*)/(*b*) solvation model, (*c*)/(*d*) crystal QM/MM and (*e*)/(*f*) protein QM/MM. Difference measures are given in Table 2 ▸. By definition, the interaction density is **P**/**C**/**S** minus **G**. Similar representations at a higher isovalue are presented in Fig. S6, which highlights that the major effect is located in the carboxylate groups that form the strongest intermolecular interactions.

Similar to our investigations in Kleemiss *et al.* (2021*b* ▸), we define the interaction density as the difference between the electron density in a given environment and the electron density of the same molecule with the same geometry without any environmental influence. The integrated number of electrons inside the grid of the interaction density, as well as the real-space *R* value *R*_RS_, are given in Table 2 ▸ and isosurfaces are shown in Fig. 5 ▸.

The shapes of the isosurfaces are remarkably similar across both substances in all environments (Fig. 5 ▸). In particular, around the carboxylate and methyl groups, the same kind of polarization is observed, *i.e.* a stronger accentuation of the lone-pair regions (electron gain) or depletion of the H atoms (electron loss). This shows that hydrogen bonding is accounted for in all environments. It is only in the carbon/silicon-exchanged position that a major difference appears: in sila-ibuprofen, there seem to be only very small effects on the electron-density distribution with respect to the hydridic H and the Si atoms, while the tertiary C atom in ibuprofen is polarized in a similar way to all other C atoms in other positions.

The highest total interaction density is found in the crystal environment in both cases (Table 2 ▸). This is not surprising, taking into account the magnitude of the electric fields of many millions of V m^−1^ inside crystals (Meyer, 1968 ▸; Dunlap & Kenkre, 1986 ▸; Fu & Cohen, 2000 ▸). However, the magnitudes of the polarization inside the enzyme and in solution are similar; all models show a significant influence of the environment on the drug molecule, with the solvation model showing the smallest effect. This is reflected in the *R*_RS_ values, as well as in the shifted number of electrons, *N*_e_ (Table 2 ▸).

To comment on the differences between ibuprofen and sila-ibuprofen, one has to take into account that ibuprofen has a total of eight fewer electrons com­pared to sila-ibuprofen. Nevertheless, it shows a higher number of shifted electrons in all environments when com­pared to its silicon equivalent. This coincides with the qualitative observation in Fig. 5 ▸ that the interaction density around the Si atom is relatively small.

### 3.3. Interaction electrostatic potential

**Figure 6:**
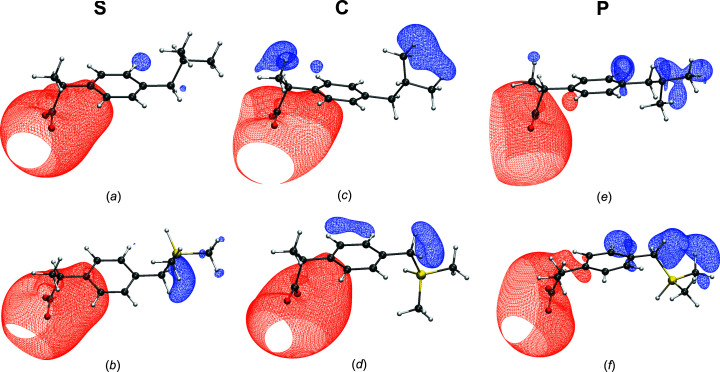
Plots of the interaction electrostatic potential isosurfaces at isovalue 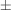 0.028 e Å^−1^ (blue = positive and red = negative) for ibuprofenate [parts (*a*), (*c*) and (*e*)] and sila-ibuprofenate [parts (*b*), (*d*) and (*f*)] in different environments: (*a*)/(*b*) solvation model, (*c*)/(*d*) crystal QM/MM and (*e*)/(*f*) protein QM/MM. Difference measures are given in Table 3 ▸. By definition, the interaction electrostatic potential is **P**/**C**/**S** minus **G**.

A com­parison of the bonding situations using a variety of com­plementary bonding indicators showed in a previous study that the atomic charges and bond properties in sila-ibuprofen, especially in the vicinity of the Si atom, are significantly different to the corresponding position in ibuprofen (Kleemiss *et al.*, 2020 ▸). Also, a significant difference was observed in the electrostatic potential of both substances because of the umpolung of the C/Si—H bond (Kleemiss *et al.*, 2020 ▸). This difference in electrostatic potential, as well as the change of direction in the dipole moment, suggest a significant difference in the response of the two drug molecules when influenced by an environment. This assumption is supported by the difference of the interaction densities in the corresponding regions of the molecules, as shown in the previous paragraph. To elucidate further, the interaction electrostatic potential was calculated in the same manner as the interaction density. Two wavefunctions – one containing the effect by the environment and a second wavefunction without any environment – were calculated and their electrostatic potential plotted in the same spatial region. The difference was then obtained by sub­trac­ting the grid of the vacuum/gas phase model (**G**) calculated using the geometry from the one that is actually experiencing the environment (models **S**/**C**/**P**). The individual plots of the interaction electrostatic potentials for both molecules are shown in Fig. 6 ▸ and the *R*_RS_ values are summarized in Table 3 ▸.

Qualitatively, a similarity in changes of potential can be observed: the areas affected are similar between ibuprofen and sila-ibuprofen. The polarization of the potential in the protein pocket in Figs. 6 ▸(*e*) and 6 ▸(*f*) has a similar direction as the crystal and solvation models. The carboxylate group becomes more negative, reflected by red isosurfaces, while the aliphatic/dimethylsilyl group becomes more positively charged. This coincides with the observed accentuation of the lone pairs of the carboxyl function in the interaction density (com­pare with Fig. 5 ▸). Again, as in the density, no direct effect around the silane functional group is observed; only the neighbouring methyl and methylene groups are affected by the environments directly.

It is observed in both substances that the effect of the environment, especially around the methyl groups of the right-hand side of the molecules in Fig. 6 ▸, is smallest in the solvation model. The crystal environment shows the highest values of *R*_RS_ (Table 3 ▸), while the surfaces around the aliphatic chain and the arene function are more pronounced in the protein systems. The higher *R*_RS_ in the crystal system is most likely due to the stronger polarization of the carboxylate groups. This is reflected by the size of the respective isosurface, which around the carboxylate functions is most pronounced in the case of the crystal models (see Fig. 6 ▸).

### 3.4. Bond-centred difference density

**Figure 7:**
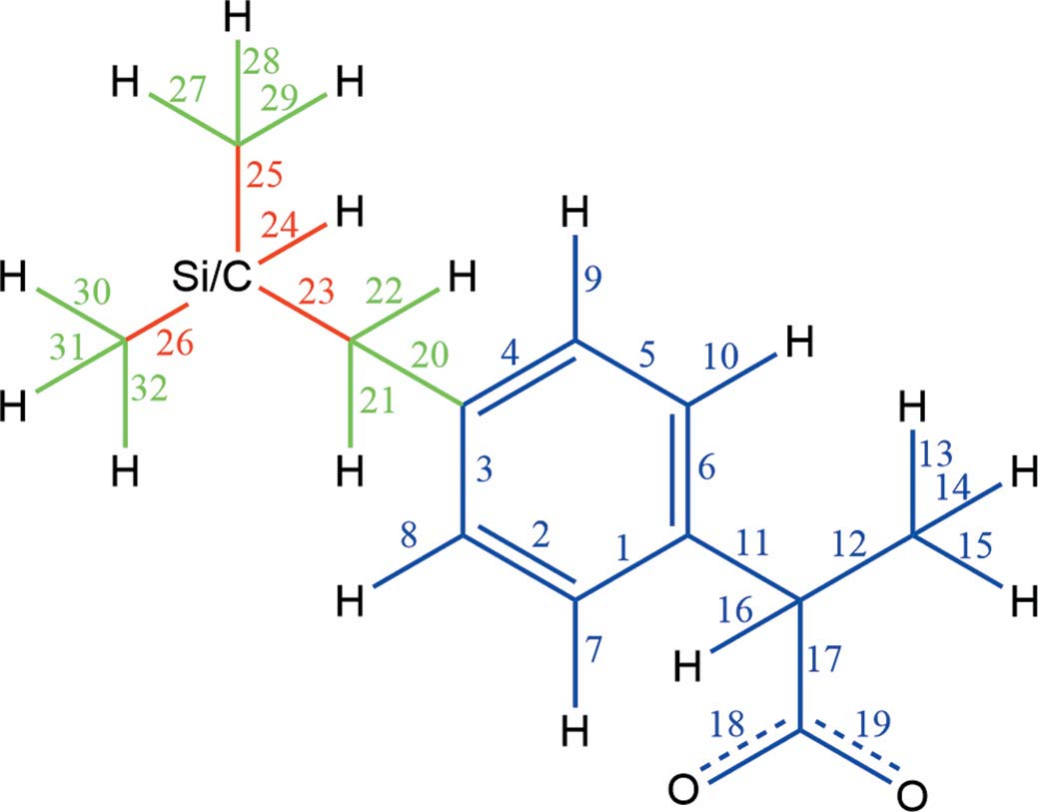
Scheme of bonds investigated in the bond-centred approach and their corresponding labels and colour scheme for later reference. Ibuprofen and sila-ibuprofen follow the same scheme and the Si/C switch is shown in the figure at the corresponding position.

To quantify the difference between the different environments in their native geometries, the bond-centred density calculation, which was introduced previously (Kleemiss *et al.*, 2021*b* ▸), was used for all bonds in ibuprofen and sila-ibuprofen. Since the definition of the calculation regions and iteration over all bonds would have been tedious and time-consuming, an automatic calculation, naming and sorting of grids, was implemented in *cuQCT* to conveniently calculate all necessary bonds with the setup of a single input file. All bonds that are found in ibuprofen and sila-ibuprofen, and their respective assigned number for the following analyses are given in Fig. 7 ▸.

**Figure 8:**
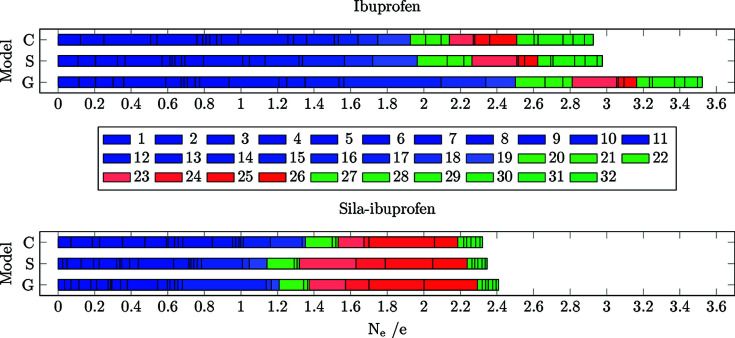
Bar plots of the cumulative integrated difference density (*N*_e_) in e between an environment (**C**, **S** and **G**) and the protein model (**P**) for all covalent bonds present in ibuprofen (top) and sila-ibuprofen (bottom). The colour code is also visualized in Fig. 7 ▸ and explained in the text.

**Figure 9:**
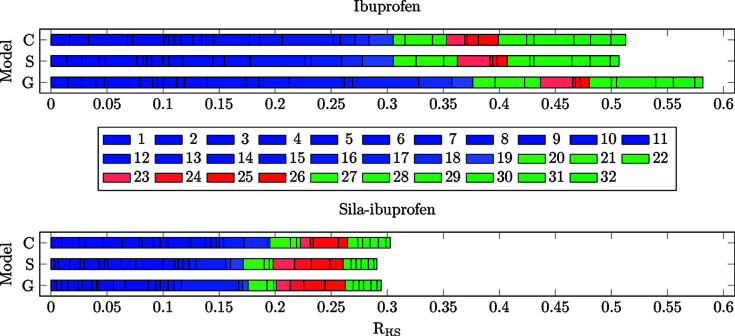
Bar plots of the cumulative *R*_RS_ values for all covalent bonds present in ibuprofen (top) and sila-ibuprofen (bottom).

Differences of the electron density were calculated for all models always referenced to the protein environment (**C**/**S**/**G** minus **P**, in contrast to the previous sections, where model **G** was the reference) and analysed in terms of the two descriptors *R*_RS_ and *N*_e_. The cumulative integrated difference electron density between two environments (*N*_e_) in each bond is shown in Fig. 8 ▸. The cumulative *R*_RS_ value of all bonds is visualized in Fig. 9 ▸.

The number of shifted electrons is highest in the gas phase in both substances (Fig. 8 ▸). The solvation model reduces this difference to the protein environment, in the case of ibuprofen, by 0.7 e. In sila-ibuprofen, this effect is much smaller, only reducing the difference by 0.1 e. This is interesting, since overall the differences between the environments in sila-ibuprofen are much smaller than in ibuprofen (*i.e.* sila-ibuprofen is less polarized by the environment), although sila-ibuprofen has an additional eight electrons when com­pared to ibuprofen and forms more polar bonds. One explanation for this observation might be the effect of the umpolung, which polarizes bonds in the sila-ibuprofen molecule internally that are less or even unpolarized in ibuprofen in the absence of an environmental influence (Laidig & Bader, 1990 ▸; Whitten *et al.*, 2006 ▸). The intramolecular dipole of the silicon–hydride bond introduces a source of polarization of the surrounding bonds in sila-ibuprofen that is not present in ibuprofen. Also, the difference in electronegativity between carbon and silicon might polarize the surrounding C atoms. Since the Si atom will donate partial electron density into the neighbouring C atoms, it can be expected that these will also influence the neighbouring H atoms in secondary effects. This would mean that bonds 20–22 and 27–32 could already show a polarization effect in the gas phase due to this intramolecular polarization. If this was the case, these bonds would most likely respond less to an external influence, since the intramolecular effects persist through all environments and are probably stronger than external environmental influences. To address this point, a summary of Quantum Theory of Atoms in Molecules (QTAIM) charges (Bader, 1990 ▸) of the corresponding atoms is shown in Table 4 ▸.

The significant magnitudes of the charges in sila-ibuprofen are due to the electronegativity difference between carbon and silicon, *i.e.* the QTAIM charges confirm that sila-ibu­pro­fen is inherently significantly more polarized than ibuprofen. In contrast, the absolute differences between the charges in any of the models with an environmental effect (**S**, **C** or **P**) com­pared to the unpolarized model (**G**) are almost always higher in ibuprofen. For example, the differences of the sum of all charges of the methyl group labelled CH_3_-1 are approximately 0.05 e in ibuprofen, while the change in sila-ibuprofen is in the range 0.012–0.025 e. Only in the solvation model does ibuprofen show a slightly lower average change in charges com­pared to sila-ibuprofen. In contrast, there is a remarkable difference in model **P** of sila-ibuprofen, where, on average, the charges change the least com­pared to model **G**. Keeping this intramolecular influence of the Si atom in mind, it is worth mentioning that the surrounding bonds of silicon in sila-ibuprofen show much smaller *N*_e_ values in the plot in Fig. 8 ▸. In other words, it is remarkable that the differences between the charges in sila-ibuprofen are so small between the continuum and explicit models although the absolute values of the QTAIM charges are large. To more deeply understand the effect of this influence of silicon, the molecule is partitioned into three different regions:

(1) bonds with a switched silicon/carbon position as a bond­ing partner; unique bonds (23–26; red colour);

(2) bonds in the vicinity of this position; one bonding partner directly bound to the carbon/silicon-exchanged position (20–22 and 27–32; green colour);

(3) bonds further away (remaining bonds 1–19; blue colour)

**Figure 10:**
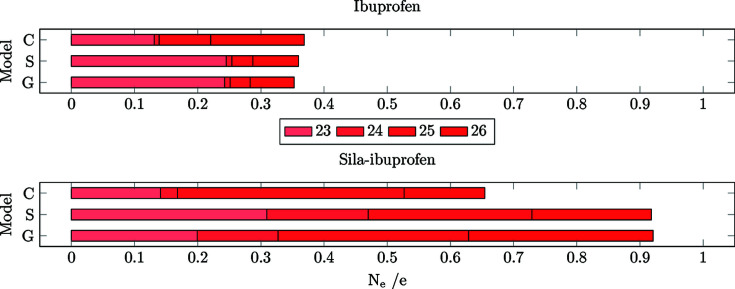
Bar plots of the cumulative *N*_e_ for the bonds 23–26 of ibuprofen (top) and sila-ibuprofen (bottom).

**(1) Bonds unique to ibuprofen and sila-ibuprofen (23–26).** The plots of *N*_e_ and *R*_RS_ for the unique bonds around the carbon/silicon-exchanged position in ibuprofen/sila-ibuprofen are shown in Figs. 10 ▸ and 11 ▸. Only looking at the *N*_e_ descriptor, the difference seems to be much larger in the case of sila-ibuprofen. The number of shifted electrons in these bonds accounts already for about a third of the total shift of electrons. In the case of ibuprofen, the difference is less than 15% of the total effect in the molecule. The reason for this is the higher number of electrons inside the region spanned by bonds 23–26 (eight more electrons in sila-ibuprofen) and the fact that there is a difference in the charge of the Si atom across the different environments, which is in the order of magnitude of 0.025 e, whereas there is practically no difference for the corresponding C atom (com­pare with Table 4 ▸). This difference of charges will also be included in the grids, even multiple times if the atom is involved in more than one bond. In this case, since silicon is involved in all bonds, the charge difference in silicon will be accounted for almost four times. Also, the sizes of the grids are significantly greater in sila-ibuprofen, since the Si—C bond length and the C—C bond length differ by almost 30%, which is almost 0.45 Å. Since the bond-scaled method also incorporates neighbouring atoms, this significantly higher integrated difference electron density might also be due to the inclusion of more effects of neighbouring atoms, due to the bigger box size when calculating the absolute integral of the differences.

**Figure 11:**
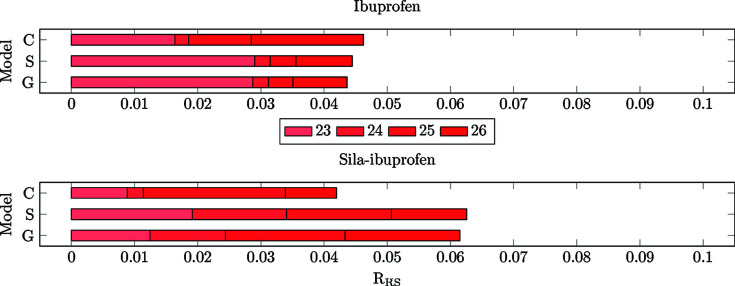
Bar plots of the cumulative *R*_RS_ values for the bonds 23–26 of ibuprofen (top) and sila-ibuprofen (bottom).

The *R*_RS_ can be understood as a normalized difference measure, since the difference is divided by the local value of the density. This allows a better com­parison among different elements (Fig. 11 ▸). In com­parison to the *N*_e_ descriptor in Fig. 10 ▸, where the bars of sila-ibuprofen are about twice as long as those of ibuprofen, the normalization in *R*_RS_ leads to a different picture. The differences are on a quite similar scale between the two com­pounds, while in the case of the crystal model, the difference for sila-ibuprofen is even smaller than that of ibuprofen. While in ibuprofen, especially in the case of models **S** and **G**, bond 23 is the most polarizable, the polarization is more evenly distributed in sila-ibuprofen.

**Figure 12:**
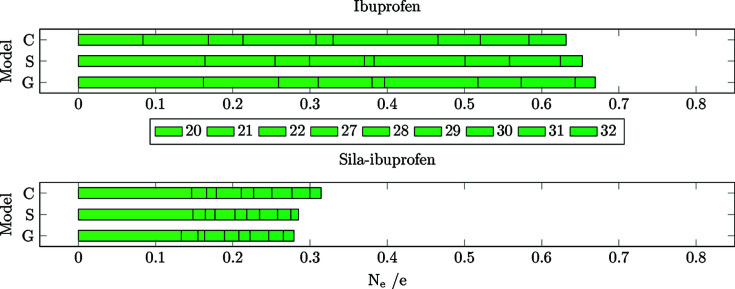
Bar plots of the cumulative *N*_e_ for the bonds in vicinity of the carbon/silicon-exchange position in ibuprofen (top) and sila-ibuprofen (bottom).

**(2) Bonds in the vicinity of the unique bonds (20–22 and 27–32).** To investigate whether the hypothesis of less polarizable bonds in sila-ibuprofen around the Si atom holds, the bond-wise differences for those bonds with one of the C atoms around the tertiary carbon/silicon position are plotted in Figs. 12 ▸ and 13 ▸. Here, the immediate impact of the different electron number is not present, only the different inherent intramolecular polarization of the vicinity of the switched/umpoled atom.

A clear difference in *N*_e_ is observed between ibuprofen and sila-ibuprofen. In sila-ibuprofen, the *N*_e_ of all the bonds is approximately half of the corresponding bonds in ibuprofen. This clearly confirms the hypothesis that more polar bonds are less polarizable. The highest polarization of all bonds in this set is observed for bond 20, which is the C—C bond directly next to the aromatic system. This bond is most likely affected by the delocalized ring system, not by substitution, and is therefore easier to polarize, since the effect is present in both molecules.

**Figure 13:**
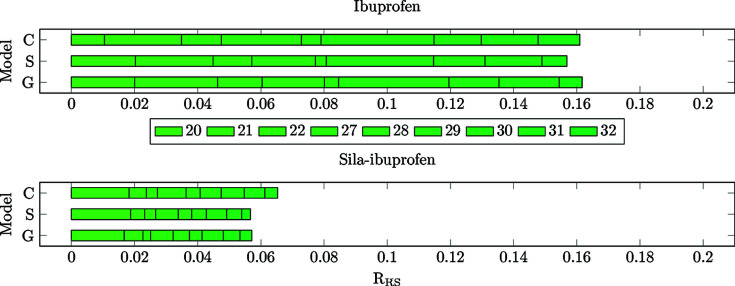
Bar plots of the cumulative *R*_RS_ values for the bonds in vicinity of carbon/silicon exchange position in ibuprofen (top) and sila-ibuprofen (bottom).

To check whether the trend is persistent when normalized for the local density, the *R*_RS_ is shown in Fig. 13 ▸. In this set of bonds, the *R*_RS_ is not as highly affected as in the set of unique bonds, and therefore the same trend as in the plots of the *N*_e_ is observed. This means that the bonds in the vicinity of the element-switched position are in fact less polarizable in sila-ibuprofen, since the effect of the environment is very small for all bonds in all environments, especially in com­parison to ibuprofen itself.

**Figure 14:**
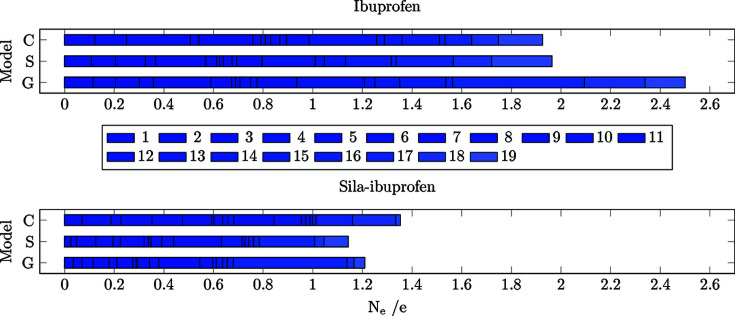
Bar plots of the cumulative *N*_e_ for the common bonds present in ibuprofen (top) and sila-ibuprofen (bottom).

**(3) Remaining bonds (1–19).** To see whether the effect of the self-polarization decreases with further distance from the switched-atom position, the remaining bonds further away from the C/Si position are shown in Figs. 14 ▸ and 15 ▸. Interestingly, the difference in polarization through the environments is also lower in all environments in the case of sila-ibuprofen for the remaining bonds. In the case of the gas phase com­parison to the protein, the effect is only half of the polarization that ibuprofen experiences. This illustrates how the effect of the umpolung by elemental substitution is not only a local phenomenon but can have long-range effects. It is imaginable that this is due to the dipole present in the molecule, as the Coulombic influence is proportional to *r* ^−2^, while other effects like dispersion have a much steeper decrease, with terms of *r* ^−6^ or even smaller exponents.

Interestingly, bond 17, which is the C—C bond connecting the carboxylate group, is the one most affected by different environments. This might be understood in terms of stabilization or destabilization of the conjugated electron system in the O—C—O group and the consequently occurring charge shift from the tertiary C atom next to it into the system. The C—C bond becomes more and more similar to the situation found in the protein going from **G** over **S** to **C**.

**Figure 15:**
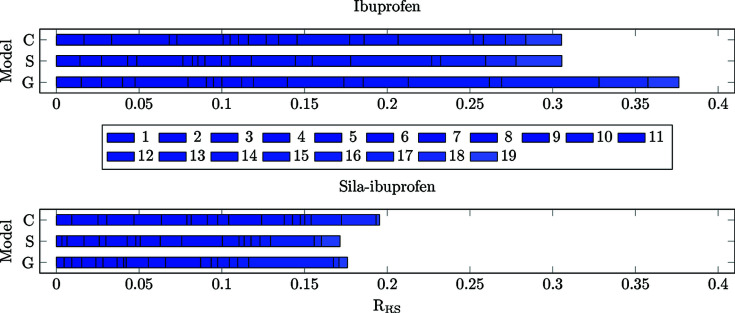
Bar plots of the cumulative *R*_RS_ values for the common bonds present in ibuprofen (top) and sila-ibuprofen (bottom).

In an antiparallel trend, the sum of *N*_e_ of both carboxylate C—O bonds 18 and 19 increases in sila-ibuprofen and ibuprofen from **G** to **S** and **C**. These trends are persistent when normalized to the local density, as was done for *R*_RS_ and which is shown in Fig. 15 ▸. The reason for the two carboxylate C—O bonds being exceptions is most likely that they are involved in the strongest and most directed intermolecular interactions of all the bonds, namely hydrogen bonds, represented by blue discs in the NCI plots in Figs. 3 ▸ and 4 ▸. These hydrogen bonds differ between the crystal packing and the enzyme environment in that there are two separate ammonium N—H⋯O18/19 hydrogen bonds with two different PEA cations in the crystal packing for both ibuprofen and sila-ibuprofen (Fig. 3 ▸), whereas there is a carboxylate–guanidinium hydrogen-bonded ring motif inside COX-II (Fig. 4 ▸; see also Fig. S6 in the supporting information).

## 4. Conclusions

In summary, sila-ibuprofen is already inherently polarized com­pared to ibuprofen – as if there was an oriented external electric field acting upon ibuprofen (Shaik *et al.*, 2016 ▸; Sowlati-Hashjin & Matta, 2013 ▸). This study has shown that sila-ibuprofen is therefore less polarizable through external influences. In this sense, the silicon/carbon switch or, in general, elemental substitution, might present the possibility to fine-tune a molecule to withstand higher environmental effects with less response of the molecular electron density. This might provide a tool to make other known organic or metal–organic molecules less prone to polarizing effects to keep a certain shape or distribution of the density in place and produce other bioisosters of known drugs.

Additionally, it was shown and quantified that the crystal is the best possible model to mimic the polarization of a molecule in the environment of the active site of the protein that it targets in biological media. This effect has been observed before for model com­pounds of protease inhibitors (Kleemiss *et al.*, 2021*b* ▸; Mladenovic *et al.*, 2009 ▸), but it is presented here for a fundamental and widely used drug. This confirms the idea of not only structural but also density and molecular properties being best understood from a crystal structure to make predictions for the situation in drug applications (Luger, 2007 ▸. However, the effect is smaller for ibuprofen/sila-ibuprofen than for the protease inhibitors investigated before in Kleemiss *et al.* (2021*b* ▸) and Mladenovic *et al.* (2009 ▸).

## Supplementary Material

Crystal structure: contains datablock(s) ibuamin, silaamin, ibu_arg. DOI: 10.1107/S2052520621009379/px5037sup1.cif

Structure factors: contains datablock(s) ibuamin. DOI: 10.1107/S2052520621009379/px5037ibuaminsup2.hkl

Structure factors: contains datablock(s) silaamin. DOI: 10.1107/S2052520621009379/px5037silaaminsup3.hkl

Structure factors: contains datablock(s) ibu_arg. DOI: 10.1107/S2052520621009379/px5037ibuargsup4.hkl

Tables S1-S7 and Figs. S1-S6. DOI: 10.1107/S2052520621009379/px5037sup5.pdf

CCDC references: 2034508, 2034509, 2034510

## Figures and Tables

**Table 1:** Crystallographic, measurement and refinement details of ibuprofen and sila-ibuprofen PEA salts after HAR using *NoSpherA2*

Structure	Ibuprofen–PEA	Sila-ibuprofen–PEA
Formula	C_8_H_12_N^+^·C_13_H_17_O_2_^−^	C_8_H_12_N^+^·C_12_H_17_O_2_Si^−^
Space group	*P*2_1_2_1_2_1_	*P*2_1_2_1_2_1_
*a* (Å)	5.9130 (12)	6.8160 (14)
*b* (Å)	15.305 (3)	12.721 (3)
*c* (Å)	22.257 (4)	23.613 (5)
*V* (Å^3^)	2014.2 (7)	2047.4 (7)
*T* (K)	25	25
*d*_max_ (Å)	0.70	0.80
λ_X-ray_ (Å)	0.3567	0.3567
*R*_int_	0.0552	0.0517
Avg. redundancy	3.82	4.15
Completeness	1.00	1.00
Average *I*/σ	29.8	14.3
No. of reflns measured	87 404	17 320
No. of unique reflns	6129	4173
Obs. criterion	*I*_o_ ≥ 2σ(*I*_o_)	*I*_o_ ≥ 2σ(*I*_o_)
No. of observed reflns	5977	2694
Weighting scheme, *w* =	1/[σ^2^(*F*_o_) + (0.0848*P*)^2^ + 2.4386*P*]^*a*^	1/[σ^2^(*F*_o_) + (0.0739*P*)^2^ + 1.1599*P*]^*a*^
No. of parameters	365	261
No. of restraints/constraints	18/6	0/27
*N*_p_/*N*_ref_	16.8	15.9
Final *R*_1_	0.0722	0.0802
Final *R*_1,all_	0.0736	0.1176
Final *wR*_2_	0.1849	0.1854
Goodness of fit	1.051	1.052
Flack^*b*^	−1 (1)^*c*^	−0.1 (4)
Max Δρ (e Å^−3^)	0.497	0.338
Min Δρ (e Å^−3^)	−0.314	−0.204
CSD deposition number	2034508	2034509

Notes: (*a*) *P* = (*F*_o_^2^ + 2*F*_c_^2^)/3. (*b*) *SHELXL* does not report the originally defined Flack parameter (Flack, 1983 ▸), but Parson’s Intensity Quotient (Parsons *et al.*, 2013 ▸). To allow comparisons with common practice, the *SHELXL*-derived parameter is reported, which cannot be compared directly to the Flack parameter reported using *olex2.refine* (Dolomanov *et al.*, 2009 ▸). (*c*) This value is unreliable, but since a defined enantiomerically purified PEA solution was added for the crystallization, the absolute configuration is known.

**Table 2:** Integrated number of electrons (*N*_e_) and real space *R* value (*R*_RS_) of ibuprofen and sila-ibuprofen over interaction density grids, visualized in Fig. 5 ▸

	Ibuprofen		Sila-ibuprofen	
Model	*N*_e_ (e)	*R*_RS_ (%)	*N*_e_ (e)	*R*_RS_ (%)
**S**	0.402	0.358	0.424	0.353
**C**	0.527	0.470	0.476	0.397
**P**	0.508	0.453	0.416	0.384

**Table 3:** Real space *R* value (*R*_RS_, %) of ibuprofen and sila-ibuprofen over interaction ESP grids, visualized in Fig. 6 ▸

Model	Ibuprofen	Sila-ibuprofen
**S**	3.178	3.126
**C**	3.853	3.421
**P**	3.769	3.232

**Table 4:** QTAIM charges in e of the atoms of the four functional groups [(C/Si)–H, CH_3_-1/2 and CH_2_] bonded to the carbon/silicon-switched position of ibuprofen and sila-ibuprofen in models **G**, **S**, **C** and **P**

	Ibuprofen				Sila-ibuprofen			
Atom	**G**	**S**	**C**	**P**	**G**	**S**	**C**	**P**
C/Si	0.101	0.100	0.101	0.100	2.764	2.768	2.793	2.789
H_C/Si_	−0.040	−0.035	−0.033	−0.041	−0.694	−0.701	−0.704	−0.694
C_\rm CH_3-1_	0.004	0.058	0.054	0.061	−0.653	−0.679	−0.673	−0.667
H_1,\rm CH_3-1_	−0.036	−0.025	−0.011	−0.024	−0.015	−0.002	−0.015	−0.018
H_2,\rm CH_3-1_	−0.038	−0.023	−0.007	−0.041	−0.021	−0.003	−0.015	−0.017
H_3,\rm CH_3-1_	−0.042	−0.027	−0.046	−0.003	−0.022	−0.003	0.007	−0.003
C_\rm CH_3-2_	0.059	0.053	0.066	0.044	−0.667	−0.681	−0.678	−0.685
H_1,\rm CH_3-2_	−0.002	−0.023	−0.047	−0.029	0.015	−0.003	−0.010	0.014
H_2,\rm CH_3-2_	−0.040	−0.027	−0.040	−0.024	−0.021	−0.004	−0.005	0.004
H_3,\rm CH_3-2_	−0.045	−0.024	−0.010	−0.005	−0.025	−0.002	−0.019	−0.019
C_{\rm CH_{2}}	0.084	0.078	0.101	0.101	−0.627	−0.617	−0.645	−0.613
H_{\rm 1,CH_{2}}	−0.038	−0.019	−0.041	−0.033	−0.025	−0.001	0.001	−0.015
H_{\rm 2,CH_{2}}	−0.042	−0.024	−0.041	−0.033	−0.028	−0.003	−0.006	−0.013
Avg. diff. to **G**	−	0.0158	0.0173	0.0186	−	0.0167	0.0167	0.0118
