# Supplementary material for: Ibuprofen and sila-ibuprofen: polarization effects in the crystal and enzyme environments
Source: Acta Crystallogr B Struct Sci Cryst Eng Mater. 2021 Nov 12;77(Pt 6):892–905. doi: 10.1107/S2052520621009379 (PMC8662945; doi:10.1107/S2052520621009379)
Supplement: Supplementary file 5 [file b-77-00892-sup5.pdf]

STRUCTURAL SCIENCE
CRYSTAL ENGINEERING
MATERIALS

Volume 77 (2021)

Supporting information for article:

Ibuprofen and sila-ibuprofen: polarization effects in the crystal and enzyme environments

Florian Kleemiss, Pim Puylaert, Daniel Duvinage, Malte Fugel, Kunihisa Sugimoto, Jens Beckmann and Simon Grabowsky

Supplementary Information for: Ibuprofen and Sila-ibuprofen - Polarization Effects in the Crystal and Enzyme Environments

Florian Kleemiss,[a,e] Pim Puylaert,[b] Daniel Duvinage,[b] Malte Fugel,[b] Kunihisa Sugimoto,[c,d] Jens Beckmann[b] and Simon Grabowsky [a]*

[a] *Universität Bern, Departement für Chemie, Biochemie und Pharmazie, Freiestrasse 3, 3012 Bern, Switzerland,* [b] *Universität Bremen, Fachbereich 2 – Biologie/Chemie, Institut für Anorganische Chemie und Kristallographie, Leobener Str. 7, 28359 Bremen, Germany,* [c] *Japan Synchrotron Radiation Research Institute / Diffraction & Scattering Division, 1-1-1 Kouto, Sayo-cho, Sayo-gun, Hyogo 679-5198, Japan,* [d] *Institute for Integrated Cell-Material Sciences (iCeMS), Kyoto University, Yoshida-Ushinomiya-cho, Sakyo-ku, Kyoto 606-8501, Japan, and* [e] *Current Address: Universität Regensburg, Fakultät für Chemie und Pharmazie, Universitätsstr. 31, 93053 Regensburg, Germany. E-mail: simon.grabowsky@dcb.unibe.ch*

A crystal of argininium ibuprofenate was measured on an in-house-diffractometer (Bruker D8 Venture, 100 K, d_{min} = 1.00 Å, for measurement details see Table S1). The crystal quality was limited and no high-resolution dataset could be obtained. The salt crystal does not contain an interaction between the carboxylate group of ibuprofenate and the arginine guanidine sidechain as in the COX environment, but instead with the ammonium function of the backbone of arginine. A visualization of the final HAR model is shown in Figure S1 and refinement statistics after HAR in *NoSpherA2* are given in Table S1. Refinement of anisotropic displacement parameters was unstable without restraints, therefore RIGU restraints were applied to all

molecules individually. The ibuprofen is not completely enantiomerically pure. Therefore, a disorder model was introduced by splitting the corresponding functional group using *SplitSAME* in *Olex2* and inverting the chirality of the second part by moving on screen. The disorder of the chiral center was refined to be 0.528(14) with higher presence of the R-enantiomer. The level of theory used for HAR was PBE/def2-TZVPP in ORCA.

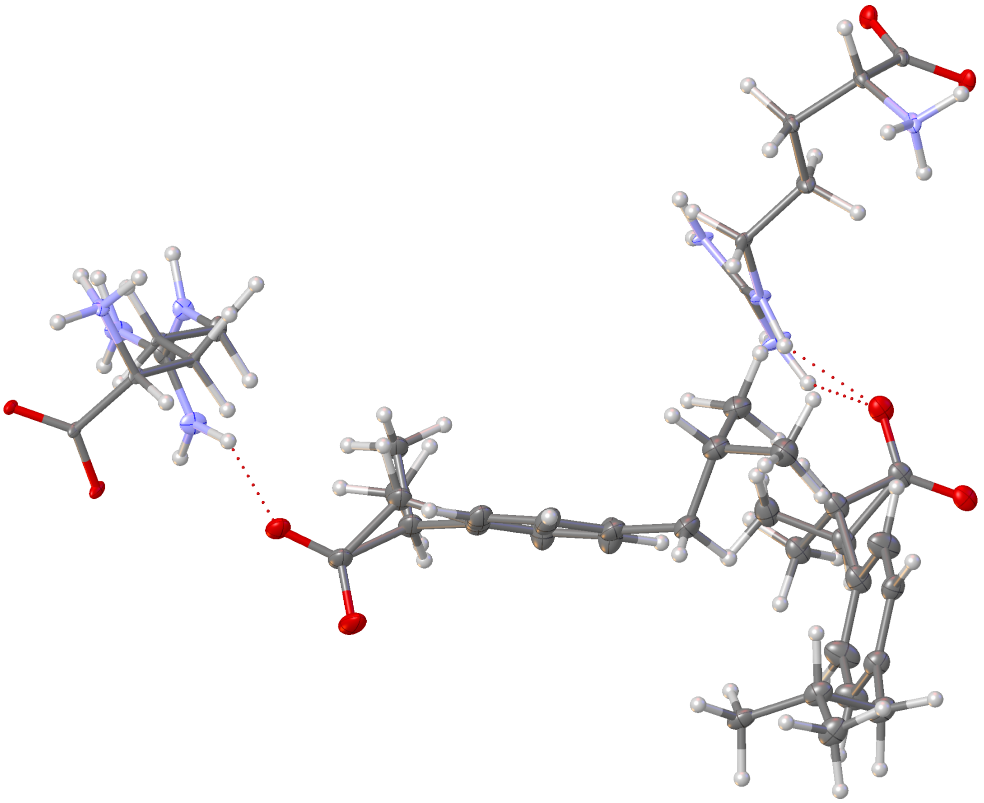

Fig. S1. Visualization of the asymmetric unit of the ibuprofen-argininium salt structure after HAR in *NoSpherA2* showing atomic displacement parameters at 50% probability level.

Table S1. *Crystallographic, measurement and refinement statistics of the ibuprofen argininium salt after HAR using NoSpherA2.*

Structure	Ibuprofen-arginine
Space group	$P\ 2_12_12_1$
a /Å	9.3033(6)
b /Å	14.8134(10)
c /Å	29.664(2)
V /Å^3	4088.1(5)
T /K	100
d_{min} /Å	1.00
$\lambda_{X\text{-ray}}$ /Å	0.71073
R_{int}	0.0552
Avg. redundancy	7.22
Completeness	1.00
Average I/σ	26.6
# of refln. measured	51814
# of unique refln.	4263
Obs. Criterium	$I_o >= 2\sigma(I_o)$
# of observed refln.	4097
Weighting scheme w=	$\frac{1}{\sigma^2(F_o)+(0.00P)^2+50.38P}$ *
# of Parameters	511
# of Restraints/Constraints	770/116
N_p/N_{ref}	8.35
Final R_1	0.1009
Final $R_{1,all}$	0.1039
Final wR_2	0.2110
Flack$^+$	-0.1(5)
Max $\Delta\rho$ /eÅ^{-3}	0.581
Min $\Delta\rho$ /eÅ^{-3}	-0.476
CSD deposition number	2034510

* $P = \frac{F_o^2+2F_c^2}{3}$; $^+$ShelxL does not report the originally defined Flack parameter, but Parson's Intensity Quotient. To allow comparisons with common practice, the ShelxL derived parameter is reported, which cannot directly be compared to the Flack parameter reported using *olex2.refine*.

Table S2. *Force field parameters for bonds (top), angles (middle) and dihedral angles used for PEA.*

Bond	Force constant (K_b) /kcal mol^{-1} Å^{-2}	Eq. distance (b_0) /Å
C_{T3} C_{T1}	222.5	1.538
H_A C_{T1}	309.0	1.111
H_A C_{T3}	322.0	1.111
N_{H3} H_C	403.0	1.040
N_{H3} C_{T1}	200.0	1.480
C_A C_A	305.0	1.3750
C_{T1} C_A	230.0	1.490
H_P C_A	340.0	1.080

Angle	Force constant (K_θ) /kcal mol^{-1} rad^{-2}	Eq. angle (θ_0) /°
C_A C_A C_A	40.0	120.0
C_{T1} C_A C_A	45.8	122.3
C_{T3} C_{T1} C_A	51.8	107.5
H_P C_A C_A	30.0	120.0
N_{H3} C_{T1} H_A	45.0	107.5
H_C N_{H3} H_C	44.0	109.5
H_C N_{H3} C_{T1}	30.0	109.5
H_A C_{T1} C_{T3}	34.5	110.1
H_A C_{T3} H_A	35.5	109.0
H_A C_{T3} C_{T1}	33.43	110.1
N_{H3} C_{T1} C_A	33.43	110.0
H_A C_{T1} C_A	33.0	109.5

Dihedral	Force constant (K_χ) / kcal mol^{-1} (K_ψ) / kcal mol^{-1} rad^{-2}	Multiplicity (n)	Eq. dihedral (δ) / °
C_A C_A C_A C_A	3.10	2	180.0
H_A C_{T3} C_{T1} C_A	0.04	3	0.0
X C_{T1} N_{H3} X	0.10	3	0.0
X C_{T1} C_{T3} X	0.20	3	0.0
X C_{T1} C_A X	0.00	6	0.0
X C_{T1} N_{H3} X	0.09	3	0.0
H_P C_A C_A C_A	4.20	2	180.0
H_P C_A C_A H_P	2.40	2	180.0
C_{T1} C_A C_A C_A	3.10	2	180.0
H_P C_A C_A C_{T1}	4.20	2	180.0

X is used as a wildcard, representing any atom type.

Table S3. *Force field parameters for Lennard Jones potentials (top) and charges of atoms in e (bottom) used for PEA.*

Atom type	ϵ /kcal mol^{-1}	R$_{min}$ /Å
C_A	-0.070	1.9924
C_{T1}	-0.020	2.2750
C_{T3}	-0.080	2.0600
N_{H3}	-0.200	1.8500
H_A	-0.022	1.3200
H_C	-0.046	0.2245
H_P	-0.030	1.3582

Atom type	Charge /e
C_{At}	-0.115
C_{Aq}	0.000
C_{T3}	-0.200
C_{T1}	0.100
N_{H3}	-0.250
H_A	0.090
H_C	0.330
H_P	0.115

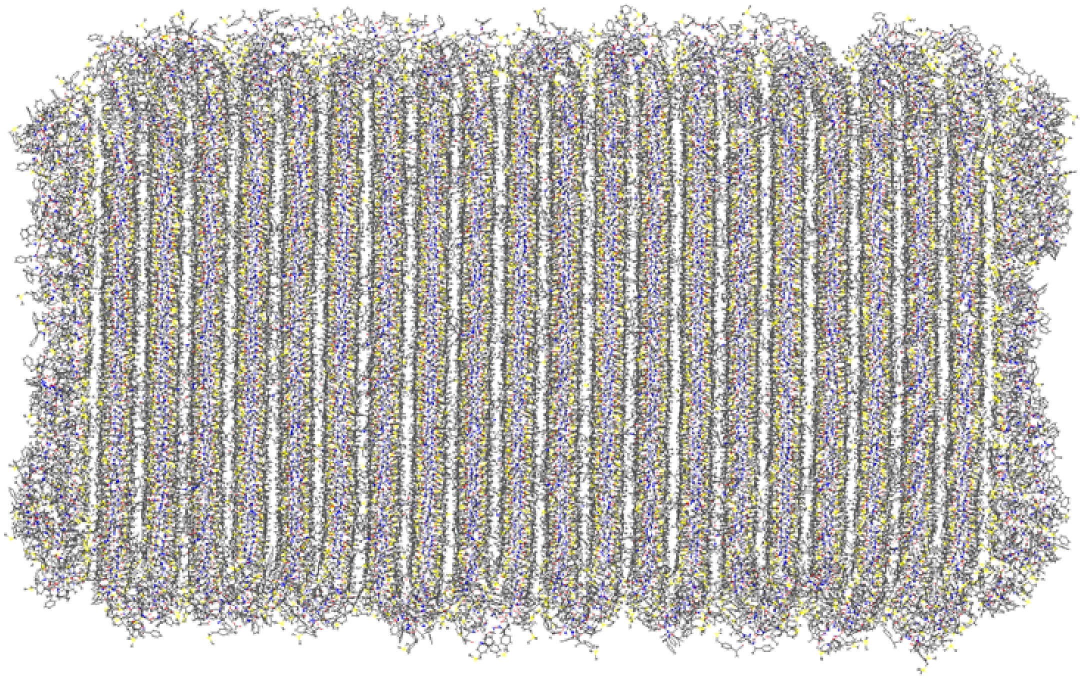

Fig. S2. Visualization of the 11x11x11 unit cell simulation box (simulation cell size of ca. 65x168x245 Å) after equilibration used for MD and QM/MM of the ibuprofen-PEA Co-crystal.

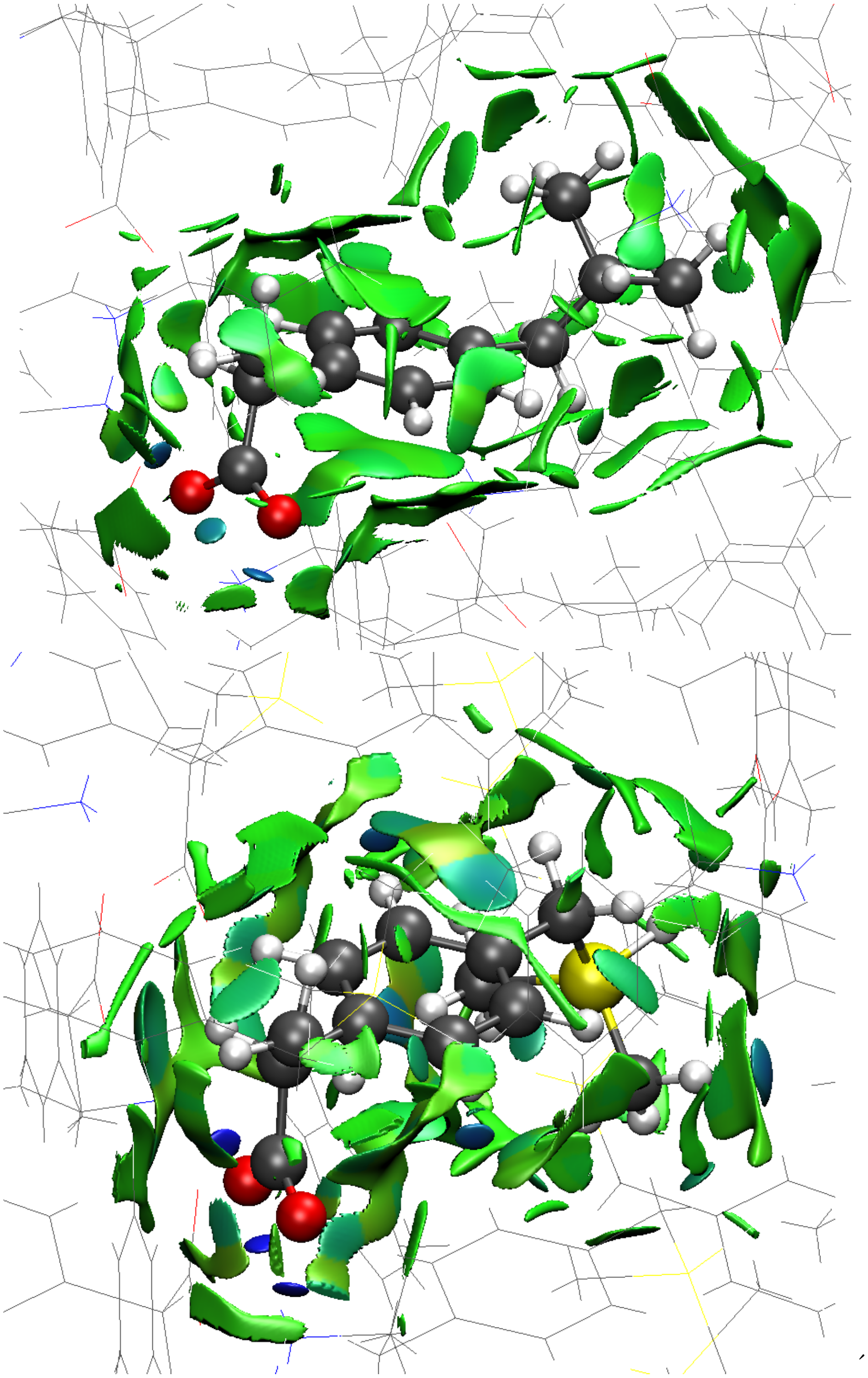

Fig. S3. Plot of isosurfaces of the NCI between ibuprofen (top) and sila-ibuprofen (bottom) and the neighboring molecules in the static crystal. Blue colored isosurfaces refer to strong (electrostatic) interactions, whereas green refer to weaker (dispersion) interactions.

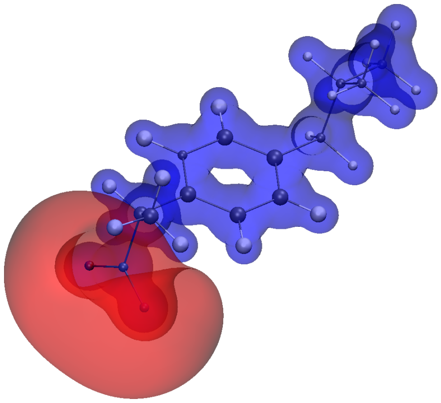

a) Ibuprofen: **S**olvation

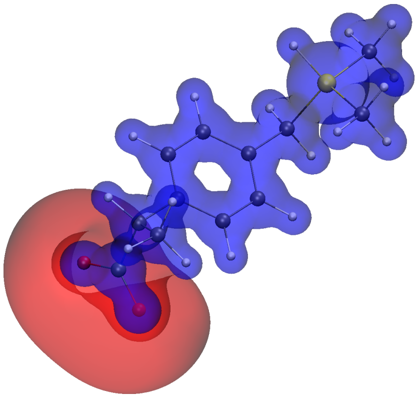

b) Sila-ibuprofen: **S**olvation

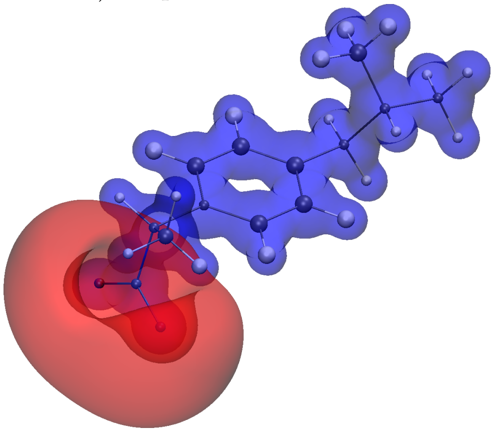

c) Ibuprofen: **C**rystal

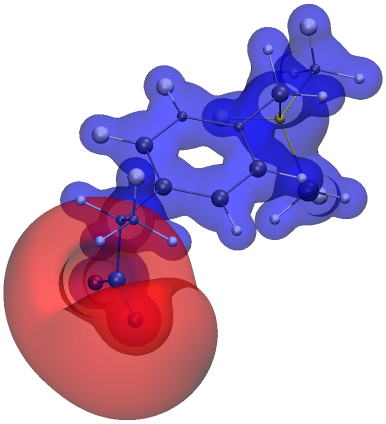

d) Sila-ibuprofen: **C**rystal

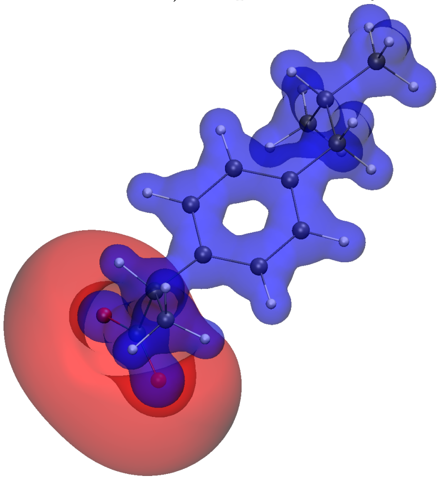

e) Ibuprofen: **P**rotein

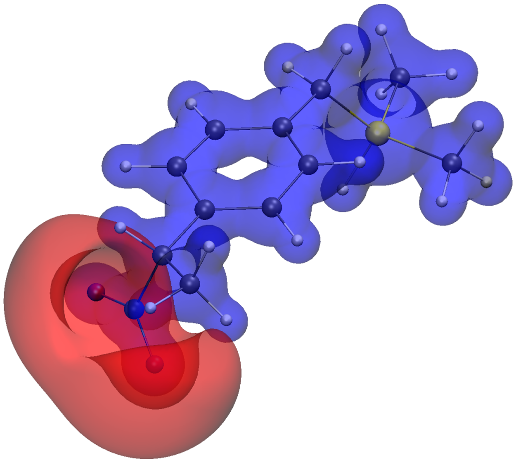

f) Sila-ibuprofen: **P**rotein

Fig. S4. Plots of the electrostatic potential isosurfaces at an isovalue ± 0.378 eÅ^{-1} (blue = positive, red = negative) for ibuprofenate (left) and sila-ibuprofenate (right) in different environments: Solvation model (a & b), Crystal QM/MM (c & d) and Protein QM/MM (e & f).

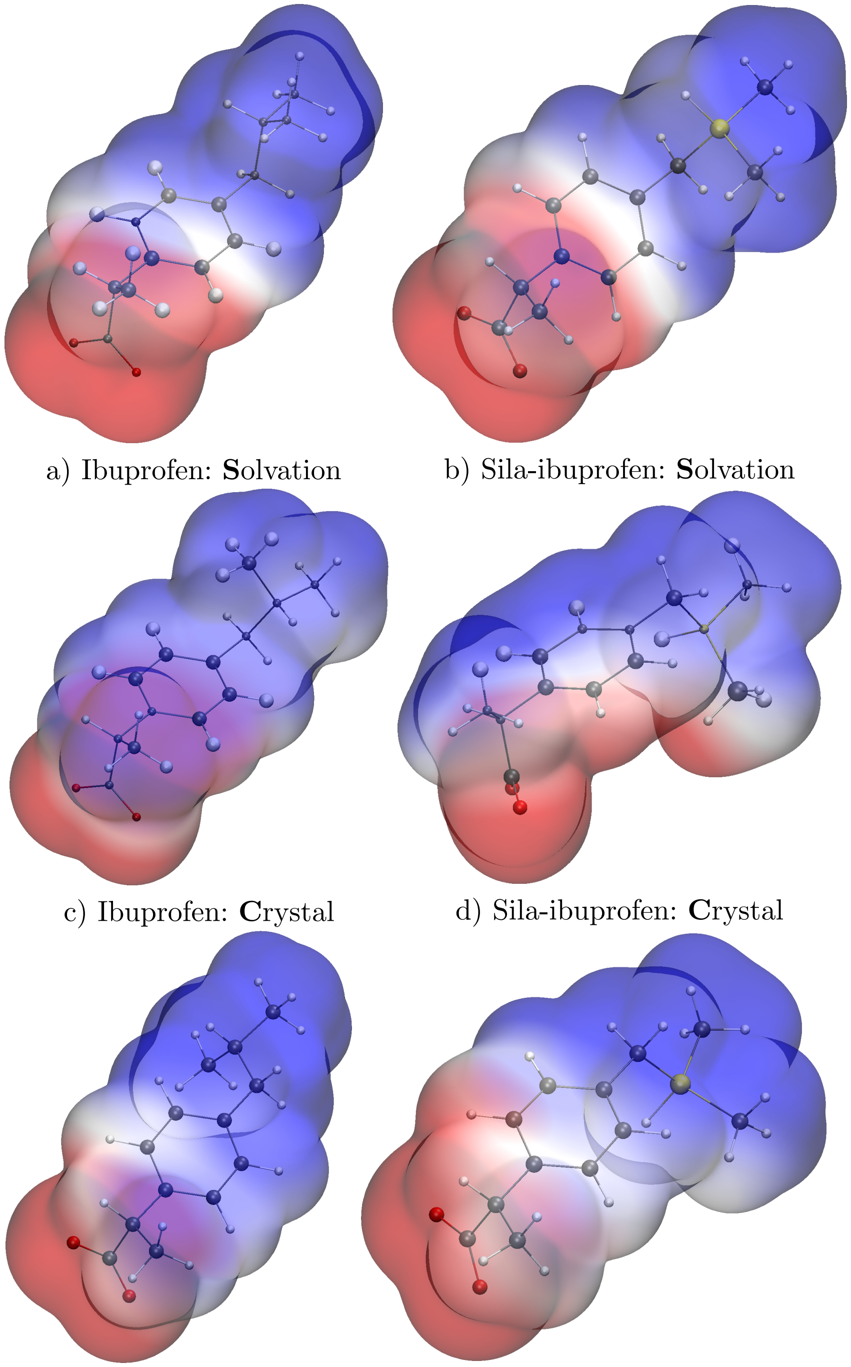

a) Ibuprofen: **S**olvation b) Sila-ibuprofen: **S**olvation

c) Ibuprofen: **C**rystal d) Sila-ibuprofen: **C**rystal

e) Ibuprofen: **P**rotein f) Sila-ibuprofen: **P**rotein

Fig. S5. Plots of the interaction electrostatic potentials mapped onto an electron density isosurface at isovalue 0.001 a.u. (blue = positive, from 0.01 a.u.; white = neutral; red = negative, up to -0.01 a.u.) for ibuprofenate (left) and sila-ibuprofenate (right) in different environments: Solvation model (a & b), Crystal QM/MM (c & d) and Protein QM/MM (e & f). By definition, the interaction electrostatic potential is **P**/**C**/**S** *minus* **G**.

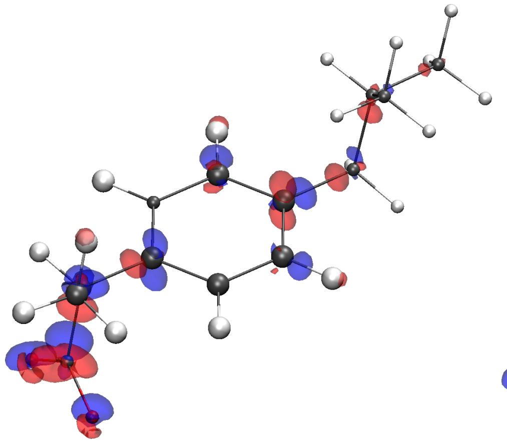

a) Ibuprofen: **S**olvation

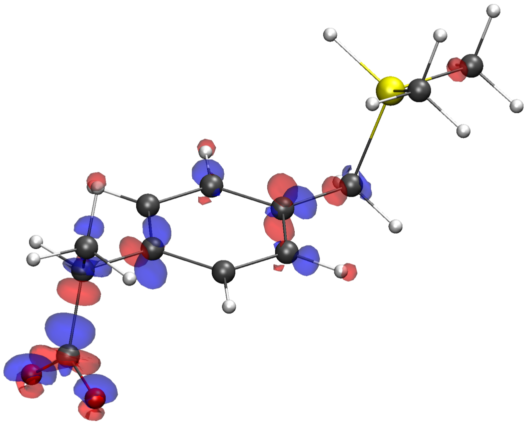

b) Sila-ibuprofen: **S**olvation

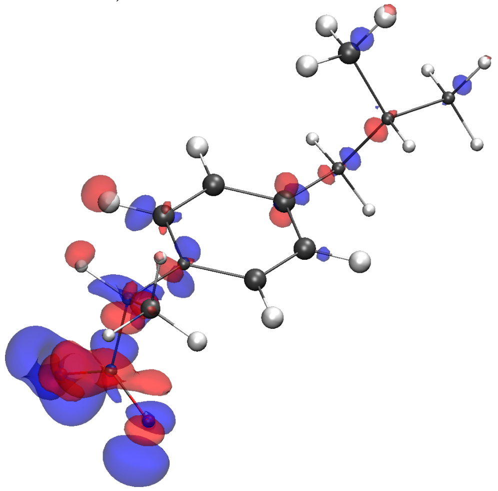

c) Ibuprofen: **C**rystal

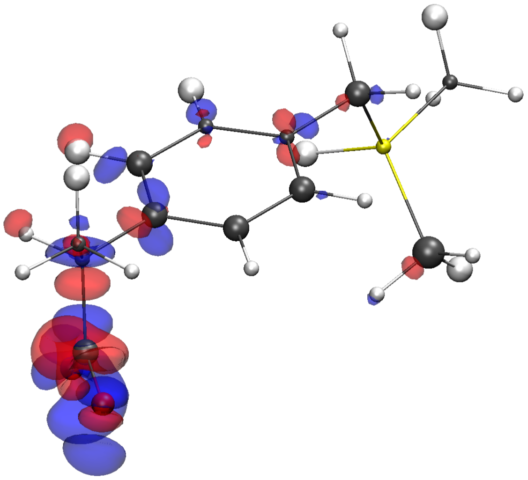

d) Sila-ibuprofen: **C**rystal

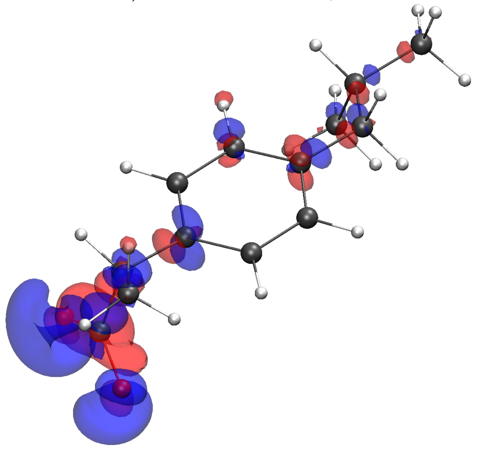

e) Ibuprofen: **P**rotein

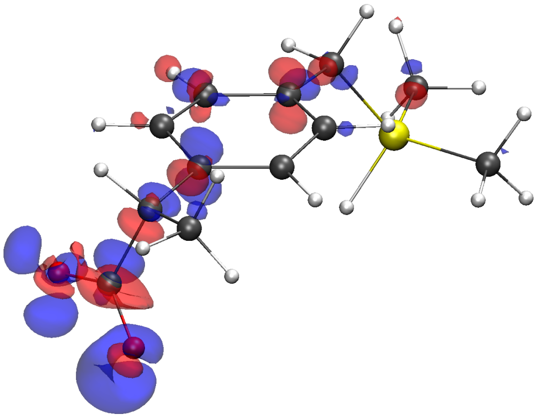

f) Sila-ibuprofen: **P**rotein

Fig. S6. Plots of the interaction density isosurfaces at isovalue ±0.003 a.u. (blue = positive, red = negative) for ibuprofenate (left) and sila-ibuprofenate (right) in different environments: Solvation model (a & b), Crystal QM/MM (c & d) and Protein QM/MM (e & f). By definition, the interaction density is **P**/**C**/**S** *minus* **G**.

Table S4. *Atomic coordinates in a.u. of isolated ibuprofenate (model G) after geometry optimization with the B3LYP/def2-TZVP level of theory inside ORCA*

Atom	X	Y	Z
C 1	6.99943401	9.00529099	8.48044315
C 2	7.94682140	6.93035809	9.81221417
H 3	9.86249368	6.30469858	9.47440202
C 4	6.48439432	5.65599475	11.59248870
H 5	7.29491480	4.06070180	12.59566026
C 6	4.01324722	6.39190394	12.12418923
C 7	3.06787752	8.48533879	10.81348370
H 8	1.16547430	9.14432486	11.20830696
C 9	4.51753143	9.75661189	9.04087613
H 10	3.77701461	11.41744331	8.10537985
C 11	10.73537996	9.03792874	5.43427825
H 12	12.22787174	8.75540201	6.82507808
H 13	10.14716083	7.20479564	4.67111665
H 14	11.55998548	10.14024150	3.89758929
C 15	8.53473670	10.48968988	6.60378270
H 16	7.25227625	11.11367781	5.11291914
C 17	2.40140990	4.99707553	14.02165516
H 18	3.61519836	3.84337082	15.23957540
H 19	1.46734922	6.36584763	15.26310608
C 20	0.33999396	3.27654201	12.87621678
H 21	-0.80114402	4.44957810	11.60887040
C 22	1.46557572	1.12188696	11.31014071
H 23	2.59582366	-0.13564014	12.50172563
H 24	-0.02231736	-0.01276572	10.43559645
H 25	2.68877645	1.83935704	9.81644801
C 26	-1.39948893	2.24806699	14.94799156
H 27	-2.27448173	3.77941486	16.02091917
H 28	-2.91135752	1.08342218	14.15729534
H 29	-0.34144145	1.07411920	16.28221544
C 30	9.46296313	13.06676014	7.92955093
O 31	11.65147259	13.03053389	8.79184519
O 32	7.84525785	14.78023255	7.93801004

Table S5. *Atomic coordinates in a.u. of ibuprofenate in a water solvation model (model S) after geometry optimization with the B3LYP/def2-TZVP level of theory inside ORCA*

Atom	X	Y	Z
C 1	6.93826439	9.20437839	8.61695398
C 2	7.95115064	7.31306269	10.15723053
H 3	9.93914347	6.85171696	10.02305073
C 4	6.44941236	6.01583105	11.88411806
H 5	7.29836910	4.56095904	13.05036798
C 6	3.87795797	6.55068346	12.14929589
C 7	2.87043744	8.45338481	10.62078178
H 8	0.88612202	8.93638775	10.78637609
C 9	4.36731228	9.74821436	8.89123406
H 10	3.52446145	11.21365306	7.73479810
C 11	10.15939866	8.97320409	5.05202222
H 12	11.55660945	7.93461548	6.14924617
H 13	8.97863543	7.61551692	4.04040735
H 14	11.17459843	10.10299814	3.65546859
C 15	8.54454546	10.67449640	6.74880177
H 16	7.25263471	11.75497739	5.56110039
C 17	2.22936098	5.13143541	13.99607686
H 18	3.42275814	4.15178135	15.37171437
H 19	1.08926947	6.48932079	15.05962209
C 20	0.41674698	3.18466530	12.79050636
H 21	-0.71674802	4.18474309	11.37647419
C 22	1.83756987	1.05030811	11.45256079
H 23	2.99946684	-0.00867359	12.79607735
H 24	0.51672287	-0.27446920	10.58107737
H 25	3.08040931	1.76828672	9.97264404
C 26	-1.38678103	2.12762650	14.79028074
H 27	-2.46479794	3.63499256	15.69850724
H 28	-2.72913469	0.80118128	13.95399879
H 29	-0.34339080	1.12017236	16.26318353
C 30	10.16562844	12.65642180	8.20356210
O 31	12.27407529	11.96125999	9.04044215
O 32	9.20999510	14.81907213	8.46538889

Table S6. *Atomic coordinates in a.u. of isolated sila-ibuprofenate (model S) after geometry optimization with the B3LYP/def2-TZVP level of theory inside ORCA*

Atom	X	Y	Z
C 1	7.09733599	9.16500963	8.45925489
C 2	7.95711595	7.08006158	9.83470445
H 3	9.83745279	6.35549141	9.49624124
C 4	6.45960911	5.92426640	11.66401009
H 5	7.21049679	4.32231962	12.70284381
C 6	4.03388862	6.79748434	12.20784928
C 7	3.16820407	8.88942871	10.84170181
H 8	1.29924311	9.63885767	11.22900270
C 9	4.65989397	10.04335627	9.02253136
H 10	3.98324389	11.70917829	8.04804238
C 11	10.80271198	8.94782903	5.38405936
H 12	12.29025753	8.61844703	6.76968459
H 13	10.11821951	7.13179630	4.66102669
H 14	11.67010706	9.97672040	3.82030802
C 15	8.68733269	10.53177256	6.53929123
H 16	7.42564871	11.18897295	5.04487985
C 17	2.39199777	5.51813119	14.16227419
H 18	3.57310973	4.51427655	15.53400967
H 19	1.32566679	6.92406279	15.24478030
Si20	0.04772603	3.17716348	12.82233672
H 21	-1.76731916	4.52969143	11.14817528
C 22	1.66397645	0.64505909	10.91979100
H 23	2.95474391	-0.46220435	12.09177148
H 24	0.28897596	-0.65322110	10.09123913
H 25	2.76669223	1.47375867	9.38766008
C 26	-1.83321141	1.68459103	15.47042852
H 27	-2.83060542	3.11679546	16.57320121
H 28	-3.24316820	0.36222974	14.74388465
H 29	-0.60437878	0.64292226	16.76366728
C 30	9.75717397	13.08203503	7.80658722
O 31	11.94575603	12.94750161	8.65961594
O 32	8.23230638	14.87841953	7.78851613

Table S7. *Atomic coordinates in a.u. of sila-ibuprofenate in a water solvation model (model S) after geometry optimization with the B3LYP/def2-TZVP level of theory inside ORCA*

Atom	X	Y	Z
C 1	7.01429378	9.46127320	8.63828045
C 2	7.92261266	7.49216428	10.15215979
H 3	9.87517939	6.90988253	9.97074196
C 4	6.37880280	6.29570699	11.90922227
H 5	7.15308292	4.78107060	13.05063376
C 6	3.85314800	7.00995902	12.24463666
C 7	2.95214075	8.99021648	10.74697679
H 8	1.01103846	9.60855618	10.95798163
C 9	4.49976753	10.18640924	8.98836975
H 10	3.73497944	11.71823482	7.86176225
C 11	9.87495131	8.97137924	4.82688060
H 12	11.20586983	7.71609561	5.76934238
H 13	8.43716683	7.83533970	3.87302273
H 14	10.92560516	10.01446792	3.38811063
C 15	8.68083254	10.79210032	6.73092734
H 16	7.48904390	12.13976912	5.72053567
C 17	2.15999655	5.66550218	14.09032858
H 18	3.26760875	4.89028438	15.65501167
H 19	0.80364196	6.98725837	14.91924500
Si20	0.25394925	2.97030913	12.67568083
H 21	-1.28532743	4.04917760	10.57063855
C 22	2.36441669	0.43690866	11.36238144
H 23	3.52006794	-0.39552847	12.85835085
H 24	1.24389203	-1.07329909	10.51270667
H 25	3.63261459	1.18149395	9.91579714
C 26	-1.95107059	1.63681934	15.12301373
H 27	-3.21662033	3.09359452	15.85586883
H 28	-3.11287335	0.12973154	14.32243181
H 29	-0.89767470	0.84849923	16.71339592
C 30	10.71017021	12.40705727	8.16048467
O 31	12.80962770	11.36207068	8.58184208
O 32	10.05926950	14.58970009	8.78660815
